# Draft Genome Sequences of 19 Clinical *stx*-Harboring Escherichia coli O80:H2 Strains

**DOI:** 10.1128/MRA.00033-21

**Published:** 2021-03-18

**Authors:** Marc J. A. Stevens, Nicole Cernela, Andrea Müller, Magdalena Nüesch-Inderbinen, Roger Stephan

**Affiliations:** a Institute for Food Safety and Hygiene, Vetsuisse Faculty, University of Zurich, Zurich, Switzerland; b Swiss National Center for Enteropathogenic Bacteria and Listeria (NENT), University of Zurich, Zurich, Switzerland; University of Maryland School of Medicine

## Abstract

Shiga toxin-producing Escherichia coli (STEC) O80:H2 is an uncommon hybrid pathotype that has emerged in Switzerland and France. Here, we report the draft genome sequences of 19 *stx*-harboring Escherichia coli O80:H2 strains isolated between 2003 and 2019 from patients in Switzerland.

## ANNOUNCEMENT

Shiga toxin (Stx)-producing Escherichia coli (STEC) strains are important foodborne pathogens and responsible for gastrointestinal illnesses which may involve nonbloody or bloody diarrhea, hemorrhagic colitis (HC), and hemolytic uremic syndrome (HUS) (1). STEC O80:H2 has recently emerged in France and Switzerland and is associated with severe cases of HUS, as well as HUS associated with bacteremia (2–5). These 19 *stx*-harboring E. coli O80:H2 strains were isolated by the National Center for Enteropathogenic Bacteria and Listeria (NENT) at the Swiss Reference Laboratory for STEC between 2003 and 2019 from stool samples (cultivation on MacConkey agar at 37°C for 24 h) of different patients in Switzerland. The presence of *stx* genes was initially determined by real-time PCR (LightCycler R 2.0 instrument; Roche Diagnostics Corporation, Indianapolis, IN, USA) using the method provided by the European Union (EU) Reference Laboratory.

The strains were grown on sheep blood agar at 37°C overnight prior to genomic DNA isolation using the DNA blood and tissue kit (Qiagen, Hombrechtikon, Switzerland). The DNA was prepared using a Nextera DNA Flex sample preparation kit (Illumina, San Diego, CA, USA), which produces transposome-based libraries that were sequenced on an Illumina MiniSeq sequencer. Up to 16 sequencing libraries were analyzed on one lane, and demultiplexing was performed using the GenerateFASTQ option in the MiniSeq local run manager v2.4.1.

The sequencing outputs were between 660,155 and 1,578,245 paired-end reads of 150 bp, resulting in a genome coverage of 37× to 89×. The Illumina read files passed the standard quality checks using the software package FastQC v0.11.7 (Babraham Bioinformatics, Cambridge, UK) and were assembled using the SPAdes v3.13.1-based software Shovill v1.0.4 (6, 7), using default settings. The assembly was filtered, retaining contigs of >500 bp, and annotated using the NCBI Prokaryotic Genome Annotation Pipeline (8). Multilocus sequence typing (MLST) was performed using the tool MLST v2.11 (https://github.com/tseemann/mlst) and the PubMLST database (https://pubmlst.org/) (9). Virulence factors were identified with VirulenceFinder v2.0 using the E. coli database (10). Stx types were determined by an *in silico* PCR using the perl script in_silico_pcr (https://github.com/egonozer/in_silico_pcr) with the option “-m, allow one mismatch” and the primers described in the EU Reference Laboratory for E. coli manual for *stx* gene detection (11). All software and databases were updated in October 2020, and default parameters were used for all *in silico* analyses.

The draft genome sequences of the 19 STEC strains consist of 5,140,014 to 5,604,512 bp divided over 204 to 361 contigs with an *N*_50_ length between 71,463 and 121,369 bp (Table 1). The GC content of the genomes is between 49.5 and 49.7 mol%. The genomes harbor 5,206 to 5,770 genes (Table 1), as predicted by the NCBI Prokaryotic Genome Annotation Pipeline. All strains belonged to the MLST sequence type 301, carried *stx*_2a_ or *stx*_2d_, the rare *eae* variant *eae*-ξ (xi), and several virulence genes typically associated with pS88 linked to extraintestinal pathogenic E. coli (ExPEC) (12). These genome sequences of clinical strains will improve the data set of the so-far limited number of sequenced STEC O80:H2 strains.

**TABLE 1:** Characteristics of the sequences of the 19 *stx*-harboring Escherichia coli O80:H2 strains

Strain	Yr of isolation	No. of reads^*a*^	Coverage (×)	Length (bp)	No. of contigs	*N*_50_ (bp)	*L*_50_	No. of genes	*stx* subtype	GenBank accession no.
1384-03	2003	1,130,346	63	5,374,262	244	90,858	17	5,432	*stx*_2a_	JAEAMT000000000
1970-08	2008	953,415	53	5,365,261	271	90,740	17	5,474	*stx*_2a_	JAEANL000000000
P17-291	2017	827,772	45	5,472,126	306	78,353	20	5,607	*stx*_2d_	JAEANK000000000
S18-168	2018	660,155	39	5,140,014	274	83,959	19	5,206	*stx*_2d_	JAEANJ000000000
S18-215	2018	669,907	37	5,500,247	284	95,494	17	5,633	*stx*_2a_	JAEANI000000000
S18-73	2018	974,815	54	5,423,481	361	88,087	19	5,598	*stx*_2d_	JAEANH000000000
S18-9-1	2018	715,345	39	5,462,066	240	121,369	15	5,585	*stx*_2d_	JAEANG000000000
S19-101-1	2019	1,402,728	78	5,419,919	314	78,353	20	5,568	*stx*_2d_	JAEANF000000000
S19-18-1	2019	1,021,583	55	5,604,512	328	71,463	23	5,770	*stx*_2a_	JAEANE000000000
S19-2-2	2019	1,578,245	86	5,503,883	301	91,161	20	5,617	*stx*_2a_	JAEAND000000000
S19-30-1	2019	673,226	38	5,321,563	287	82,429	20	5,430	*stx*_2d_	JAEANC000000000
S19-615-1	2019	1,006,838	55	5,489,739	342	91,272	19	5,649	*stx*_2d_	JAEANB000000000
S19-64-1	2019	739,020	40	5,505,413	284	95,454	19	5,611	*stx*_2a_	JAEANA000000000
S19-677-1	2019	911,398	50	5,444,745	342	95,454	18	5,583	*stx*_2a_	JAEAMZ000000000
S19-710-1	2019	837,499	45	5,576,432	318	79,701	22	5,706	*stx*_2d_	JAEAMY000000000
2017-299-1	2017	991,420	56	5,329,337	310	73,469	19	5,460	*stx*_2d_	JAEAMX000000000
2017-353-1	2017	791,789	43	5,531,847	361	78,109	20	5,701	*stx*_2d_	JAEAMW000000000
2018-226	2018	922,198	50	5,508,417	290	94,354	20	5,647	*stx*_2a_	JAEAMV000000000
2018-439	2018	1,211,877	70	5,183,983	272	75,540	21	5,265	*stx*_2d_	JAEAMU000000000

a Refers to the number of reads in one set of the paired-end 150-bp reads.

### Data availability.

This whole-genome shotgun project has been deposited at DDBJ/ENA/GenBank under the accession no. JAEAMT000000000 to JAEANL000000000. The versions described in this paper are version no. SAMN16883199 to SAMN16883217. The raw sequencing reads have been deposited in the SRA under the BioProject accession no. PRJNA680568.
